# *In vitro* and *in vivo* models for androgenetic alopecia drug development

**DOI:** 10.1242/dmm.052848

**Published:** 2026-07-08

**Authors:** Shan Tu, Ayaka Nanmo, Yuki Migita, Tomoki Asaba, Jieun Seo, Sayuri Hamano, Tatsuto Kageyama, Junji Fukuda

**Affiliations:** ^1^ Faculty of Engineering, Yokohama National University, 79-5 Tokiwadai, Hodogaya-ku, Yokohama, Kanagawa 240-8501, Japan; ^2^ Institute of Advanced Science, Yokohama National University, 79-5 Tokiwadai, Hodogaya-ku, Yokohama, Kanagawa 240-8501, Japan; ^3^ Kanagawa Institute of Industrial Science and Technology, 3-2-1 Sakado Takatsu-ku, Kawasaki, Kanagawa 213-0012, Japan

**Keywords:** Androgenetic alopecia, Drug screening, Hair follicle models

## Abstract

Alopecia is a common disorder that can cause hair loss owing to various factors, including genetics, stress and diet. Androgenetic alopecia (AGA), also known as male pattern baldness, is the most common type of progressive hair loss. AGA symptoms can be alleviated by effective drugs, such as finasteride and minoxidil. Therefore, most patients with alopecia select drug therapy as the first-line treatment. However, their side effects and variable efficacy have increased the demand for alternative treatment options for these patients. Despite the high clinical demand, the development of novel therapeutic agents remains limited, primarily due to the lack of physiologically relevant drug testing models that accurately predict drug effects in humans. In this At a Glance article, we provide an overview of the currently available *in vitro* models for hair drug testing. First, we outline the limitations of traditional models, such as two-dimensional cultures and animal models, in reproducing hair follicle structures and multicellular signaling pathways. We then introduce three-dimensional culture platforms that partially overcome these drawbacks. Next, we discuss the differences and applications of various models in terms of functional readouts, reproducibility, high-throughput potential, standardization and cost. We highlight recent progress in the development of follicular skin model constructs and propose practical metrics for selecting the most appropriate model for the initial screening of potential hair drugs. Overall, this At a Glance article provides a roadmap towards a more translationally valuable screening system for the discovery and development of hair loss treatments.

## Teaser

**Summary:** The lack of physiologically relevant hair follicle models hinders the understanding of skin biology and the development of effective treatments for hair disorders. This At a Glance article provides an overview of existing *in vitro* hair follicle models, outlining their advantages, limitations and future perspectives.

**Figure:**
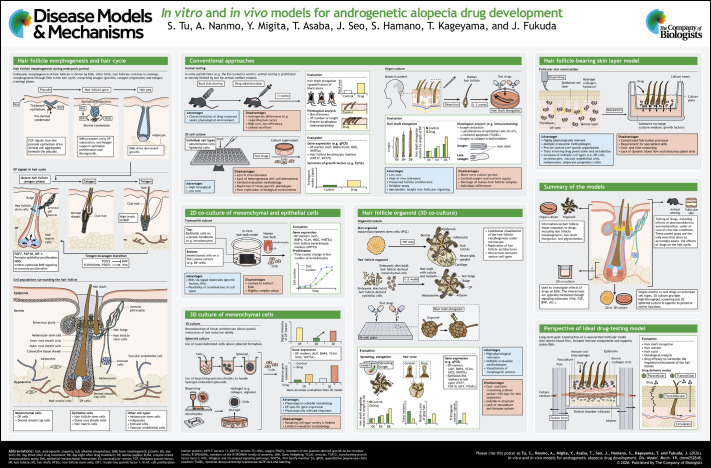
See supplementary information for a high-resolution version of the poster.

## Introduction

Androgenetic alopecia (AGA), the most common form of alopecia, affects ∼80% of men up to the age of 80 years (York et al., 2020). Its prevalence in the male population varies by region, with high rates reported in the USA (41.2%) and Europe (35%) (Vañó-Galván et al., 2019). Generally, AGA is less common in Asian men compared with European males and men the USA, with rates of 14.1 and 21.3% reported in South Korea and China, respectively (Paik et al., 2001). However, Pathomvanich et al. (2002) demonstrated that the prevalence of AGA in Thailand is 38.5%, comparable to that in Europe and the USA. The prevalence of AGA in women increases with age, ranging from 3−12% in Caucasian women aged 30–40 years (Starace et al., 2020). Regional variation in AGA prevalence is possibly driven by a combination of racial, genetic and environmental factors, including diet (Ambra et al., 2025).

The hair follicle (HF) is a mini-organ that repeatedly generates the hair shaft during the hair cycle (see poster ‘Hair follicle morphogenesis and hair cycle’). The hair cycle in the human scalp consists of three main phases: anagen (growth; 2–7 years), catagen (regression; 2–3 weeks) and telogen (rest; 3–4 months). The entire cycle lasts 2–7 years, during which hair grows ∼1–1.5 cm/month in the anagen phase (Grymowicz et al., 2020; Oh et al., 2016; Schwartzenfeld and Karamikian, 2010). AGA is closely associated with progressive shortening of the anagen phase and follicular miniaturization (Inui and Itami, 2011). Mechanistically, elevated androgens (such as dihydrotestosterone) bind to androgen receptors in the HF, altering key signaling networks, such as downregulation of the Wnt/β-catenin pathway and upregulation of the bone morphogenetic protein (BMP) pathway, which prematurely drives the HF into regression. Over the past two decades, various potentially active molecules have been screened for the treatment of AGA. These include androgen receptor antagonists, Wnt and sonic hedgehog signaling (SHH) molecule agonists, BMP inhibitors, 5α-reductase inhibitors, thyroid hormone receptor activators and mitochondrial pyruvate carrier inhibitors (Kim et al., 2025). Only 11 compounds entered clinical trials for AGA treatment between 2004 and 2024, with most failing to advance to subsequent phases and, ultimately, being discontinued (Luo et al., 2024). The only fundamental drugs used in clinics with proven safety and efficacy in many countries, including the USA, EU, Korea and Japan, are finasteride and minoxidil (Rossi et al., 2012). Finasteride is a type II 5α-reductase inhibitor, directly reducing dihydrotestosterone production, thereby suppressing AGA progression and improving its symptoms. Clinical studies have shown that oral finasteride (1 mg/day) increases hair count by 107 hairs per 5.1 cm^2^ area over 1 year (Kaufman et al., 1998). Dutasteride inhibits both type I and II 5α-reductases. Phase III trials indicated that it increases hair density by 12.2 hairs/cm² over 6 months in Asian AGA patients (Eun et al., 2010). Dutasteride has been approved in some countries, such as Japan (Tsunemi et al., 2016) and South Korea (Choi et al., 2016). However, it remains unapproved for treatment of AGA in the USA and Europe, since its safety profile remains controversial. This is primarily because it has a significantly longer half-life in the human body (Gupta et al., 2022b). In the USA and Europe, dutasteride is typically prescribed off-label, i.e “situations where a medicinal product is intentionally used for a medical purpose not in accordance with the terms of the marketing authorisation” [European Patients' Academy of Therapeutic Innovation (EUPATI), see Legal framework in Europe] for AGA treatment. Minoxidil, an ATP-sensitive potassium channel (K_ATP_ channel) opener with vasodilatory properties, enhances blood flow and indirectly improves oxygen and nutrient delivery to HFs (Choi et al., 2016; Shorter et al., 2008; Tsunemi et al., 2016). It supports the anagen phase thereby promoting proliferation and inhibiting apoptosis of the dermal papilla (DP) cells at the base of each HF. This effect is achieved through extracellular-signal-regulated kinase and phosphoinositide 3-kinase/AKT signaling, as well as through upregulation of the vascular endothelial growth factor (VEGF), which stimulates angiogenesis and activates the Wnt/β-catenin pathway to enhance HF-growth-related gene expression (Han et al., 2004; Kwach et al., 2011; Lachgar et al., 1998). Clinical studies focusing on AGA in males in the USA have shown that topical application of 5% minoxidil increases hair density by ∼20 hairs/cm² after 24 weeks of treatment (Olsen et al., 2002). However, topical application of 5% minoxidil only increased hair regrowth by 9.1 hairs/cm² in women with female pattern hair loss (Bergfeld et al., 2016). This difference likely stems from different hormonal mechanisms driving male and female hair loss. Minoxidil requires activation by follicular sulfotransferase, whose activity varies between sexes and significantly affects clinical outcomes (Roberts et al., 2014).

The U.S. Food and Drug Administration-approved drugs still exhibit some limitations. For example, neither finasteride nor minoxidil can fully reverse long-standing and end-term AGA, for which many HFs are severely miniaturized and lost. Patients with AGA sometimes complain of side effects – including persistent or irreversible sexual, neurological, physical and mental side effects, known as post-finasteride syndrome – during and/or after discontinuation of finasteride treatment (Traish, 2020). Clinical data indicate that minoxidil treatment induces skin irritation, such as scalp pruritus (severe itching) and hypertrichosis. Typical minoxidil-induced hypertrichosis refers to unwanted hair growth, most commonly on the chin, forehead and upper lip (Desai et al., 2024). Specifically, 8.8% of male patients treated with 5% minoxidil reported scalp pruritus/irritation, whereas 46.4% of female patients experienced hypertrichosis (Gupta et al., 2022a). The incidence of these side effects tends to increase with increasing minoxidil concentrations (Gupta et al., 2023).

The medical community has yet to sufficiently recognize this syndrome and no evidence-based effective treatment is currently available. Therefore, there is an urgent need to identify and evaluate alternative compounds with comparable or superior efficacy, and fewer side effects. Although several unapproved or investigational therapies exist, most lack persuasive evidence from adequately controlled trials, limiting their broader adoption and commercialization (Katzer et al., 2019). The fundamental problem is that current evaluation systems cannot reproduce morphologically and functionally intact HFs *in vitro*, and lack a unified and quantifiable readout. This bottleneck slows the drug development process, increases costs and leads to high attrition because compounds that appear effective *in vitro* and in animal models often fail to produce meaningful benefits in humans (Castro et al., 2022), wasting time and resources. Recently, alternative models, such as HF organoids, have emerged and may provide more reliable outcomes. However, dynamic vascular networks and immune cell integration are lacking in *in vitro* models, highlighting the need for more physiologically relevant platforms that better reflect HF biology (Valdez-Zertuche et al., 2025).

This At a Glance article summarizes and compares hair models, from classic approaches, such as animal experiments, two-dimensional (2D) cultures and HF organ cultures, to more recent approaches, such as 2D co-cultures, three-dimensional (3D) spheroids and HF organoids. Additionally, it highlights the importance of integrated skin–HF models with vascular–immune components for future hair drug development.

## HF biology

### HF development and hair cycle

Drug testing models often derive insights from developmental processes. *In vivo*, HFs undergo morphogenesis through epithelial–mesenchymal interaction during embryonic development (Popescu and Höcker, 2007; Sennett and Rendl, 2012; Wang et al., 2023). HF morphogenesis is initiated by hair placode formation (see poster ‘Hair follicle morphogenesis and hair cycle’). First, the cells in the upper dermis activate Wnt/β-catenin signaling. The overlying epithelial cells receive these signals and, in turn, activate their own Wnt/β-catenin and ectodysplasin A/nuclear factor-κB pathways, leading to epithelial cell thickening and formation of hair placodes (Chen et al., 2012; Mok et al., 2019). Subsequently, FGF signals from the placode epithelium drive dermal cell aggregation beneath the placode (Huh et al., 2013). The hair placodes grow downwards to form HF germs above the dermal condensates, which differentiate into the DP (Fuchs and Horsley, 2008). During this process, epithelial Wnt/β-catenin signaling activates *Shh* expression. SHH signals from epithelial cells promote their proliferation and downward growth (Abe and Tanaka, 2017). Epithelial SHH also induces noggin (NOG) production within the underlying dermal condensate, which blocks BMP signaling and, therefore, lifts BMP-mediated inhibition, enabling HF formation (Lu et al., 2016; Rishikaysh et al., 2014). As the hair germs develop into hair pegs, reciprocal epithelial–mesenchymal signaling promotes the downgrowth of the follicular epithelium into the dermis, where epithelial cells begin to surround the dermal condensate as it develops into the DP (Driskell et al., 2011; Park, 2022). Together, these signals build a layered follicle and establish sites for hair shaft elongation (Lee and Choi, 2024; Zhang and Chen, 2024). After birth, mature HFs undergo the hair cycle. During the early anagen phase, niche-derived signals activate HF stem cells (HFSCs). These cells proliferate and give rise to progenies that differentiate into the hair shaft, which continuously elongates during the anagen phase. Upon entering the catagen phase, HFs rapidly shorten due to apoptosis of the outer root sheath and extrusion of the inner root sheath. In the telogen phase, inhibitory signals keep stem cells quiescent. At telogen exit, they reactivate and a new anagen phase begins (Alonso, 2006).

### Cell types in HFs

#### Mesenchymal cells

Mesenchymal cells in HFs include DP and dermal sheath cup cells (see poster ‘Hair follicle morphogenesis and hair cycle’, subpanel ‘Cell populations surrounding the hair follicle’). DP cells are specialized mesenchymal cells located at the base of HFs, which originate from PR domain zinc finger protein 1 (PRDM1, also known as Blimp1)-positive (Blimp1+) fibroblasts during embryonic development (Ji et al., 2021). DP cells regulate follicular development and growth mainly via signaling molecules (Driskell et al., 2011). During the anagen phase, activated DP cells stimulate the proliferation of epithelial stem cells in HFs, driving downward follicle growth and DP encapsulation (Driskell et al., 2011; Ohyama et al., 2010). DP cells activate the Wnt/β-catenin pathway and regulate other pathways, including SHH, BMP and FGF pathways (Enshell-Seijffers et al., 2010; Greco et al., 2009; Silva-Vargas et al., 2005). Because DP cells direct neighboring epidermal cells to differentiate along the HF lineage (Reynolds et al., 1999), strategies that restore or enhance this inductive capacity show potential for hair loss treatment (Taghiabadi et al., 2020). Dermal sheath cup cells are located in the deepest part of the HF and differentiate into DP cells to maintain the DP.

#### Epithelial cells

Epithelial cells in the HF include HFSCs, outer root sheath cells and hair matrix cells (see poster ‘Cell populations surrounding the hair follicle’). HFSCs are a group of slow-turnover, long-term, self-renewing, multipotent stem cells located in the bulge of the outer root sheath of HFs (Cotsarelis et al., 1990). They maintain a low level of division during the rest period. Upon stimulation by signals, such as Wnt/β-catenin, BMP/transforming growth factor beta 2 (TGFβ2), Notch and SHH, these cells are activated and rapidly differentiate into transit-amplifying precursor cells that give rise to all epithelial lineages of HF cells, including keratinocytes and inner/outer root sheath cells (Legue and Nicolas, 2005; Sanchez-Danes and Blanpain, 2017). The fate of HFSCs is regulated by multiple exogenous microenvironmental signals (adipose, vascular, neural and immune cells, as well as by the matrix) that are key indicators of hair cycle initiation and transition.

Hair matrix cells are a group of epithelial cells that reside at the base of HFs, specifically in the bulb. They receive signals from the adjacent DP, proliferate, move upward, and then differentiate into multiple layers of the outgrowing hair shaft and the channel surrounding the hair shaft, known as the inner root sheath (Millar, 2002; Morgan, 2014).

#### Other cell types

HFs also contain accessory cell types, including melanocyte stem cells, adipocytes, immune cells and vascular endothelial cells, that form the HF niche (see poster ‘Cell populations surrounding the hair follicle’). Melanocyte stem cells occupy the hair bulge and differentiate into melanocytes during the hair cycle, they then migrate to the hair bulb and deposit melanin into the growing hair shaft, causing pigmentation. Defects in melanocyte stem cell maintenance underlie hair graying (Nishimura et al., 2002). Adipocytes occupy the dermal white adipose tissue that surrounds the lower HF, and their primary function is to secrete adipokines and provide metabolic support. All cells of the adipocyte lineage are derived from mesenchymal stem cells through the activation of several factors, including the BMP, Wnt and SHH signaling pathways (Kruglikov et al., 2019). Mature adipocytes inhibit hair-cycle progression, whereas non-committed adipocyte stem cells stimulate DP cells and promote HF cycling (Plikus et al., 2011). Immune cells, including skin-resident macrophages and regulatory T cells, provide regulatory signals that influence HFSC activation and differentiation (Ali et al., 2017; Castellana et al., 2014). Vascular endothelial cells form a perivascular niche that coordinates with follicular progenitors during development (Li et al., 2019). These endothelial cells assemble into capillary networks, providing blood supply, and deliver essential oxygen and metabolites to the follicle.

## Conventional drug testing models

Conventional drug testing models have provided the foundation for AGA research. These approaches mainly include animal models, 2D cell culture and *ex vivo* HF organ culture. They offer established workflows and readouts but differ markedly in terms of physiological relevance, throughput and translational predictability (Table 1).

**Table 1.** Structural and functional characteristics of each AGA drug-testing model

			Conventional models			Advanced *in vitro* models			
			2D	Mouse	Organ culture	3D	2D co-culture	HFO	HF skin
Biological accuracy	Structure	Dermal papilla	✓	✓	✓	✓	✓	✓	✓
		Hair bulb		✓	✓		✓	✓	✓
		HF		✓	✓			✓	✓
		HF+niche		✓					✓
	Functional characteristics	GF secretion (DP signal)	✓	✓	✓	✓	✓	✓	✓
		Response to DP signal of epithelial cells		✓	✓		✓	✓	✓
		Hair shaft formation		✓	✓			✓	✓
		Hair cycle		✓					
Applications	Drug screening		✓	✓	✓	✓	✓	✓	✓
	Hair regeneration			✓				✓	✓
	Mechanism study			✓	✓			✓	✓
	Safety testing			✓					

HF, hair follicle; HFO, hair follicle organoid; GF, growth factor; DP, dermal papilla.

Conventional mouse models: C57BL/6 and C3H strains.

## Animal testing

Mouse models with hair abnormalities have been widely used in research, as they provide genetic and molecular biology insights into hair growth, and facilitate the evaluation of drug responses and safety via skin absorption tests (see poster ‘Conventional approaches’). Although the anagen phase of human scalp hair (which typically lasts 2–7 years) is different from that of mouse dorsal hair (which typically lasts ∼2 weeks) (Müller-Röver et al., 2001), the short and relatively rapid hair cycle in mouse dorsal skin enables rapid and reproducible evaluation of anagen induction or growth inhibition *in vivo*. Importantly, mouse and human HFs share conserved epithelial and mesenchymal compartments and key signaling pathways that regulate hair cycling and regeneration. These characteristics make mouse HFs useful models for hair regeneration studies (Lee and Choi, 2024; Yi, 2017).

The mouse strains most commonly used as models to assess anagen induction and drug activity *in vivo* are C57BL6 (Shin et al., 2013) and C3H (Hamada and Suzuki, 1996; Sundberg et al., 1994) (Table 1). The dorsal skin of C57BL/6 mice shows relatively synchronized hair cycling, and anagen entry is accompanied by visible skin pigmentation. This feature enables the rapid visual scoring of anagen induction in depilation-based assays. C3H mice also have well-characterized hair cycling and are frequently used in hair-loss studies, which facilitates comparisons across datasets. A study by Crabtree et al. (2010) used transgenic mice overexpressing human androgen receptor and treated them with high levels of androgen 5α-dihydrotestosterone to model key features of human AGA, including delay in hair regeneration. In addition to androgen-based models, endocrine manipulation can be used to probe hair cycle control and drug responses. Topical application of estradiol to mouse skin inhibits hair growth by blocking HF transition from the resting to anagen phase, which can be reversed by estrogen receptor antagonists (Chanda et al., 2000).

While animal models are invaluable in hair-related research, they exhibit some limitations. First, interspecies variations limit the translational value of the results derived from mouse models, as the hair cycle in mice differs from that of humans (Castro et al., 2022). Second, animal experiments are time consuming – often taking weeks to months to complete – and costly due to expensive animal housing, which is subject to strict welfare rules, making high-throughput screening impractical. Finally, animal experiments require careful ethical considerations. Indeed, in some jurisdictions (e.g. the EU cosmetics sector), animal testing is prohibited or strictly limited by law for animal welfare reasons. Although animal models are necessary for the validation of systemic effects and toxicology (Shugart, 1985; Soni et al., 2017; Taškova et al., 2018), they should be complemented by *in vitro* systems that are more physiologically relevant to humans than animal models.

## 2D cell culture

Since its large-scale introduction at the end of the 20th century, 2D cell culture has remained the most common *in vitro* platform for the primary screening of compound libraries because of its operational simplicity, low cost and high-throughput compatibility (see poster ‘Conventional approaches’). In the context of hair drug screening, individual types of cell – including DP cells (Han et al., 2004; Kwack et al., 2011; Nilforoushzadeh et al., 2020), keratinocytes (Boyera et al., 2009; Zhou et al., 2018) and melanocytes (Shi et al., 2016; Tarafder et al., 2014; Yang and Boissy, 1999) – are seeded on culture plates, maintained in standardized medium and, after treatment with the target substance, and analyzed. This can be done by staining, i.e. immunofluorescence or alkaline phosphatase (ALP) staining, and molecular biology assays, i.e. quantitative polymerase chain reaction (qPCR), western blotting and enzyme-linked immunosorbent assay (ELISA). This reduces microenvironmental confounders and simplifies the interpretation of drug effects. However, it does neither capture drug penetration nor gradients present in the intact skin and HFs. Common functional metrics include cell viability, migration rate, expression of characteristic genes and their protein levels, such as alkaline phosphatase in DP cells and tyrosinase in melanocytes (Madaan et al., 2018; Tobin, 2008).

Similar to many other specialized cell types, DP cells and melanocytes gradually lose their specific abilities under 2D culture conditions, leading to significant differences in their *in vivo* behaviors and responses (Higgins et al., 2013; Ohyama et al., 2012). The 2D cell culture inherently lacks the *in vivo* 3D structure of HFs, epithelial–mesenchymal interactions and biomechanical stimulation, such as extracellular-matrix-derived stiffness and tension cues. Moreover, it is challenging to simulate the combined effects of drugs observed in the complex HF microenvironment in a 2D setting. This is mainly because niche cell types are absent, and spatial gradients and diffusion barriers are not reproduced in the model. In addition, the reproducibility of such experiments is often limited by differences in matrix coatings, serum sources and passage numbers (Baker and Chen, 2012; Langhans, 2018). Therefore, 2D cell culture is suitable for rapid primary screening and generating mechanistic hypotheses (Table 1). However, more physiologically relevant 3D models are essential to better meet clinical needs.

## HF organ culture

Complex signaling pathways regulate hair generation and growth in HF (see poster ‘Conventional approaches’; Rogers and Hynd, 2001). To study these signaling pathways *ex vivo*, intact HFs can be isolated from human scalp tissues or rat vibrissae. Rat vibrissae are often used because they are easy to isolate and relatively consistent in size, shape and structure. These isolated HFs are typically cultured individually in culture dishes using liquid medium. These methods allow maintaining an intact HF outside the body for >2 weeks, even under serum-free conditions (Haslam et al., 2018; Ohn et al., 2019; Westgate et al., 1993). Culturing isolated HFs in a serum-free environment eliminates interference from undefined hormones and growth factors present in the serum, and ensures accurate evaluation of added drugs. This approach preserves the native 3D structure, which is lost in 2D cell culture. Organ culture can be used to test specific therapeutic candidates in HFs. These candidates include small-molecule drugs, hormones and growth factors – such as 5-hydroxytryptamine, which was shown to activate DP cells and promote hair growth in human HF organ culture (Kageyama et al., 2025). This approach allows researchers to investigate intra-follicular responses that closely resemble those observed *in vivo* (Ahn et al., 2012) and is simpler and less expensive than HF reconstruction *in vitro* (Table 1), which is discussed below under ‘Hair follicle organoid culture’. A standard test to select effective drugs is the assessment of hair shaft elongation (Parodi et al., 2018), which can be achieved by determining HF keratinocyte proliferation and apoptosis through staining for the cell proliferation marker protein Ki-67 (officially known as MK167), and by using terminal deoxynucleotidyl transferase dUTP nick-end labeling (TUNEL), respectively (Kloepper et al., 2010; Langan et al., 2015).

However, HF organ culture has some limitations. Although the early stages of anagen-to-catagen transformation can be monitored under organ culture conditions (Kloepper et al., 2010), the lack of blood vessels, nerves, and immune and endocrine systems make assessment of long-term hair-cycle progression, pharmacokinetics and systemic toxicity impossible. Human HFs can typically be maintained *ex vivo* for approximately ≤3 weeks, after which viability and hair growth decline, possibly due to limited oxygen and nutrient diffusion in avascular culture (Langan et al., 2015). In addition, isolating HFs is labor intensive and time consuming, with a limited number of human samples available. Considerable variation between samples, e.g. differences in size and stage of the hair cycle, often causes deviation from experimental data.

## Tissue-engineered *in vitro* drug-testing models

Tissue-engineered models address the major limitations of conventional testing platforms. They overcome the lack of 3D architecture in planar cultures and bypass species differences inherent to animal models. The fundamental principle behind their fabrication involves isolating specific follicular cell populations and guiding their co-culture or self-assembly processes. A defining characteristic of these advanced models is their ability to recreate essential epithelial-mesenchymal interactions and faithfully mimic the native follicular microenvironment of the scalp (Table 1).

## 2D co-culture of HF epithelial and mesenchymal cells

Co-cultures of HF epithelial and mesenchymal cells are used to reproduce *in vivo* epithelial–mesenchymal interactions in HF. A typical approach involves culturing these two cell types in a spatially separated manner (see poster ‘2D co-culture of mesenchymal and epithelial cells’). For example, keratinocytes can be cultured in the upper compartment of Transwell^®^ inserts on a porous membrane, whereas DP cells are grown in the underlying well on a collagen type I-coated plate. These two cell types can continuously exchange signaling molecules via the porous membrane (typically with a pore diameter of 0.4 μm) (Kitagawa et al., 2009; Ohyama et al., 2012). Epithelial–mesenchymal interactions upregulate levels of alkaline phosphatase and lymphoid enhancer-binding factor-1 in DP cells (Veraitch et al., 2017). This upregulation indicates a restored hair-inductive capacity and characteristic follicular phenotype. Additionally, these interactions upregulate keratin differentiation markers, such as KRT75 and KRT17, in keratinocytes (Chan et al., 2015). Different cell types can be flexibly combined according to research needs, such as DP cells from the AGA scalp combined with normal keratinocytes (Kitagawa et al., 2009). Additionally, indicators of hair-inductive capacity can be read in various ways (morphology, genes and mechanics), making this approach suitable for validation of mechanisms and secondary screening of drug candidates (Edmondson et al., 2014; Langhans, 2018; Thippabhotla et al., 2019; Table 1). However, compared to 2D cell culture, the above-described cell culture setup is slightly more complex. Moreover, this co-culture system lacks epithelial–mesenchymal interactions via direct cell–cell contact, preventing the formation of proper 3D follicular structures.

## 3D culture of DP cells (spheroid culture)

3D cell aggregates have been used to generate various tissue types and are referred to as embryoid bodies when derived from embryonic stem cells, neurospheres when derived from neural cells, and spheroids when derived from other cell types. When maintained in spheroid culture, individual cells exhibit a more physiological cuboidal morphology and form denser cell–cell contacts (Baker and Chen, 2012; Higgins et al., 2013) than in 2D cell culture. DP cell spheroids can be prepared using several approaches, including the use of non-cell adhesive culture plates (Kageyama et al., 2019; Ohyama et al., 2012), hanging drop culture (Lin et al., 2016), and encapsulation in extracellular matrix hydrogels, such as Matrigel^®^ and collagen (see poster ‘3D culture of mesenchymal cells’; Tan et al., 2021). Naturally, DP cells reside as spherical aggregates in the HF bulb, which probably enables them to partially restore their DP-specific transcriptome when cultured as spheroids *in vitro* (Higgins et al., 2013), thereby leading to increased expression of trichogenic (hair growth-promoting) genes related to the Wnt/β-catenin and phosphoinositide 2-kinase/Akt signaling pathways (Lin et al., 2020). Therefore, DP spheroids are suitable models for screening hair growth-promoting compounds, such as minoxidil (Bejaoui et al., 2022). In conventional 2D cell cultures, DP cells rapidly dedifferentiate and lose their specific functions after repeated passages. By passage 8, these cells typically exhibit a complete loss of their hair-inductive capacity. Spheroid culture enables DP cells to regain many characteristics of primary DP cells even after passage 8 and enhances their ability to induce HF neogenesis *in vivo* following transplantation (Lin et al., 2016). The limitations of DP spheroids include lack of communication with epithelial cells, melanocytes and immune components, as these cell types are absent in the culture. Therefore, this approach is unsuitable for investigating the processes that involve such interactions in HFs, including hair cycle transitions and hair shaft pigmentation (Table 1). Additionally, because conventional spheroids are typically scaffold free, spheroid culture systems may exhibit particularly weak interactions with the extracellular matrix. This physical limitation can affect drug diffusion, cell responses, drug efficacy and toxicity readouts (Baker and Chen, 2012).

## HF organoid culture

Organoid-based approaches have expanded from simplified HF models to tissue reconstruction, enabling *in vitro* systems that closely mimic skin development [see poster ‘Hair follicle organoid (3D co-culture)’]. Skin organoids are generated using induced pluripotent stem cells (iPSCs) to recapitulate epithelial–mesenchymal interactions and aspects of early skin development (De Henau et al., 2025; Jung et al., 2022; Kim et al., 2024; Lee et al., 2018, 2020; Lee and Koehler, 2021; Shafiee et al., 2024), such as epidermal structures and typical HF-like appendages. Such iPSC-derived hair-bearing skin organoids have been used for drug testing (Lee et al., 2020). Researchers can apply specific therapeutic compounds to modulate the key signaling pathways. This targeted modulation visibly alters HF development and hair shaft output. Consequently, these organoids provide a direct and quantifiable readout for assessing drug efficacy (Lee et al., 2020). Furthermore, researchers have utilized these advanced organoid systems to evaluate the chemical perturbations affecting follicular fate and overall hair growth (Shafiee et al., 2024). Overall, iPSC-derived models have the potential to reconstruct the complex human skin architecture *in vitro*. However, generating mature HF structures in iPSC-derived skin organoids typically require >100 days of culture (Lee et al., 2020), which may limit their applicability to hair-focused studies and high-throughput drug screening due to time and cost constraints (Table 1).

To address these limitations, HF organoids have been developed using fetal/adult skin-derived cells (Kageyama et al., 2022a,b; Lei et al., 2017, 2023; Xie et al., 2022). As these cells can only differentiate into skin or HF lineages, HFs can be prepared much more quickly in culture. HF organoids recreate epithelial–mesenchymal interactions within HF-like structures containing epithelial, mesenchymal and melanocytes, and can produce hair shafts *in vitro* (Kageyama et al., 2022b).

Compared to conventional *in vitro* systems, HF organoids more closely resemble *in vivo* HFs, making them better platforms for drug screening and mechanistic exploration (Jang et al., 2020; Kageyama et al., 2023; Marinho et al., 2024). Researchers can directly assess the efficacy of various drug candidates by measuring the subsequent hair shaft elongation (Kageyama et al., 2023, 2024a,b, 2025). The pigmented HF structure makes organoids suitable for testing strategies aimed at addressing gray hair (Tu et al., 2025). However, limitations prevail in vascularization, immune components and overall tissue architecture , particularly, given that HF formation follows an outside–in structure, with mesenchymal cells on the outside and epithelial cells on the inside of the organoid (Kageyama et al., 2022a; Lei et al., 2017). This indicates that existing organoid systems are insufficient to fully model drug penetration and systemic responses. Future studies should focus on developing new HF-bearing skin layer models that closely mimic the structure of native hair-bearing skin.

## HF-bearing skin layer model

Drugs for hair loss are typically delivered either topically or orally, and each route reaches the target cells through different mechanisms. Topical drugs for hair loss penetrate the stratum corneum or skin pores to reach target cells, such as DP cells, in HFs (Patzelt and Lademann, 2020). Oral drugs reach target cells via the bloodstream and, in some cases, may exert effects by acting on cells outside the HFs, such as vascular endothelial cells (Gupta et al., 2023). HF responses to drugs may vary depending on the surrounding microenvironment. Key microenvironmental factors include the integrity of the epidermal barrier and the dynamic local vascular network. To better replicate such microenvironments *in vitro*, extensive efforts have been made to engineer full-thickness skin models containing HFs (see poster ‘HF-bearing skin layer model’). Bioprinting has been used to fabricate skin models with integrated epidermis, HFs and vascularized dermal components (Abaci et al., 2018; Motter Catarino et al., 2023). In such reconstructed skin models, spheroids containing human DP cells and vascular endothelial cells are bio-printed into a fibroblast-laden hydrogel that represents dermis. The cells in spheroids self-assemble into concentric follicle-like layers, and vascular endothelial cells form microvessels that emulate *in vivo* vascular exchange by delivering essential oxygen and nutrients to the local microenvironment (Abaci et al., 2018; Motter Catarino et al., 2023).

HF-bearing skin layer models are physiologically relevant platforms to evaluate trans-follicular drug delivery, efficacy and toxicity (Motter Catarino et al., 2023; Table 1). Although these *in vitro* models resemble natural tissues, they lack certain complexities of *in vivo* human skin, such as follicular sebaceous gland units, dynamic blood flow, immune regulation and endocrine signaling. Additionally, skin models remain technically demanding and expensive to construct, with long preparation times and complex workflows, further limiting their scalability and throughput.

## Conclusions and future perspectives

In conclusion, we reviewed existing hair drug-testing models, including their advantages and disadvantages, noting the trade-off between physiological relevance and throughput/cost (see poster ‘Summary of the models’). Notably, organoids and follicular skin constructs improve human relevance but remain limited by their variability and high costs. Future studies should focus on improving the accuracy of readouts (including penetration and efficacy), reducing costs through simpler standardized workflows, and developing more precise skin models that better simulate the native follicle–epidermis environment. The long-term goal should be to construct a vascularized follicular skin model that includes blood vessels and immune components, supports stable epithelial–mesenchymal interactions and shows a reproducible hair cycle. Such efforts will facilitate the testing of skin and follicular drug penetration, tracking of drug metabolism, and evaluation of the long-term effects of different drugs on hair growth, pigmentation, and inflammation, making screening more reliable and practical for hair loss drug development.

To realize these long-term goals and bridge the *in vitro*–*in vivo* gap, the integration of advanced microphysiological systems (such as hair-follicle-on-a-chip) holds great potential (see poster ‘Perspective of ideal drug-testing model’). These dynamic platforms can simulate blood flow and systemic drug exposure, thereby offering a more precise evaluation of drug pharmacokinetics, including paracellular, transfollicular and transcellular delivery dynamics, and long-term toxicity. Crucially, such advanced systems must be capable of mimicking the hair cycle to address the limitations of currently approved drugs like finasteride and minoxidil, which fail to fully reverse long-standing miniaturization in AGA. Developing these cycle-competent models allows researchers to directly assess how drug candidates prevent catagen entry or promote anagen maintenance, thereby effectively correlating *in vitro* readouts with actual clinical efficacy (such as hair shaft growth).

Furthermore, establishing unified, reproducible and quantifiable screening protocols across different laboratories is essential for translating *in vitro* findings into clinical success. In this regard, the integration of artificial intelligence (AI) into organoid and microphysiological research offers a promising approach. AI-driven image analysis and machine learning can automate cell tracking, classify cell types, and extract actionable insights from large biological datasets, ultimately accelerating the transition from experimental models to clinical applications (Liu et al., 2024; Bai et al., 2025).

## Poster

Poster
