# Supplementary figures and images for: In vitro and in vivo models for androgenetic alopecia drug development
Source: Dis Model Mech. 2026 Jul 8;19(6):dmm052848. doi: 10.1242/dmm.052848 (PMC13382705; doi:10.1242/dmm.052848)

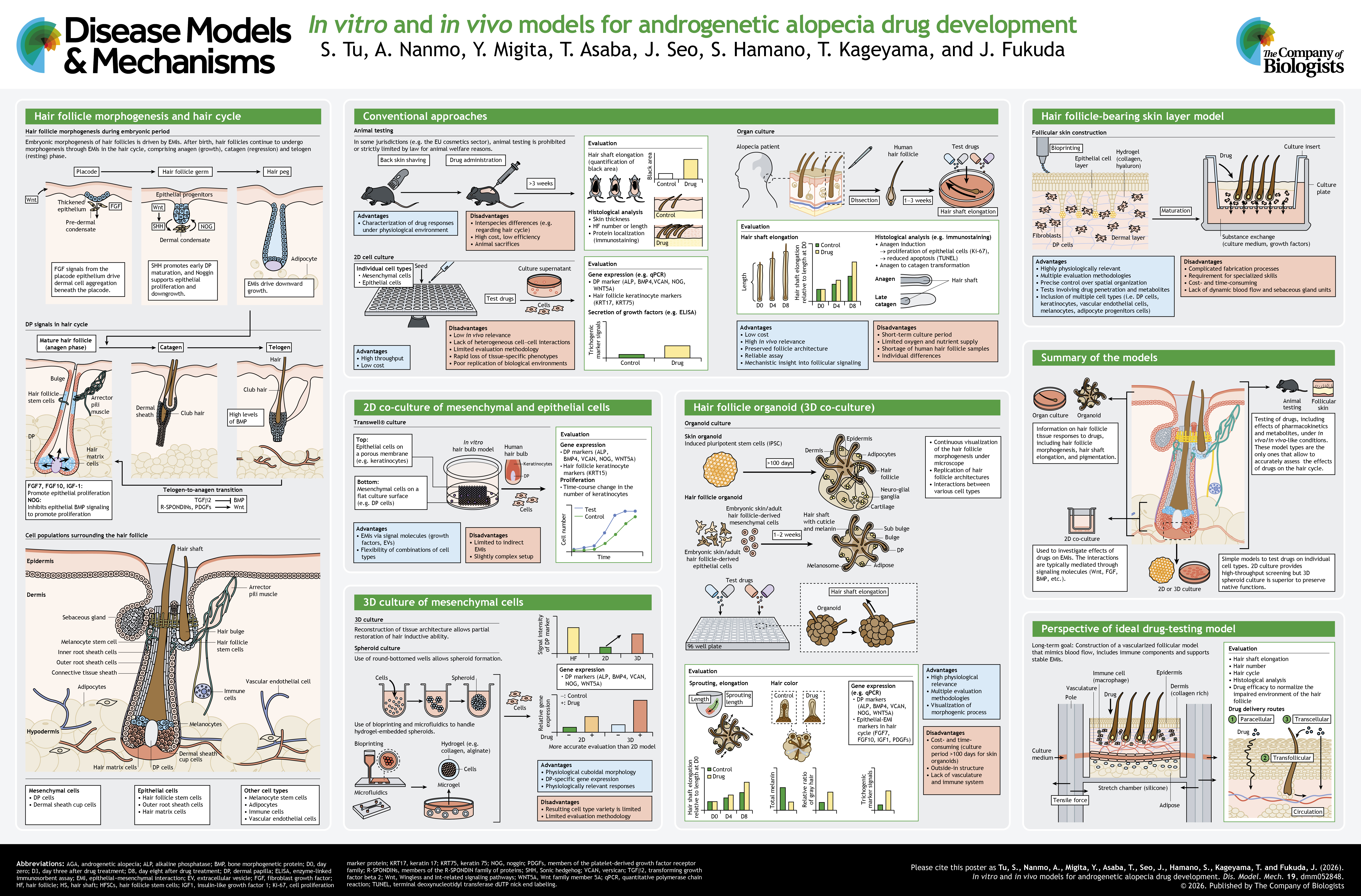

Supplement: Poster [file dmm-19-052848-s1.jpg]
